# Dietary melatonin, chronotype, and night eating syndrome in relation to sleep quality among adults

**DOI:** 10.3389/fnut.2026.1890075

**Published:** 2026-08-13

**Authors:** Selda Geylani, Yagmur Demirel Ozbek, Nurgul Arslan

**Affiliations:** 1 Department of Nutrition and Dietetics, Faculty of Health Sciences, Uskudar University, Istanbul, Türkiye; 2 Department of Nutrition and Dietetics, Faculty of Health Sciences, Recep Tayyip Erdoğan University, Rize, Türkiye; 3 Department of Nutrition and Dietetics, Ataturk Faculty of Health Sciences, Dicle University, Diyarbakır, Türkiye

**Keywords:** chronotype, circadian rhythm, dietary melatonin, night eating syndrome, sleep quality

## Abstract

**Objective:**

Dietary melatonin has attracted increasing interest because of its potential relevance to circadian regulation and sleep health. However, evidence regarding its relationship with chronotype, night eating behaviors, and sleep quality remains limited. This study aimed to examine the associations among estimated dietary melatonin intake, chronotype, night eating behaviors, and sleep quality in adults and to explore the cross-sectional indirect association among chronotype, night eating behaviors, and sleep quality.

**Methods:**

This cross-sectional study was conducted in Istanbul, Türkiye, between November 2024 and June 2025 and included 325 adults aged 18–65 years. Sleep quality was assessed using the Pittsburgh Sleep Quality Index (PSQI), chronotype using the Morningness–Eveningness Questionnaire (MEQ), and night eating behaviors using the Night Eating Questionnaire (NEQ). Dietary melatonin intake was estimated using a modified food frequency questionnaire based on melatonin-containing foods. Pearson correlation analysis, multiple linear regression, binary logistic regression, and an exploratory cross-sectional indirect-association analysis using PROCESS Model 4 were performed.

**Results:**

Mean total dietary melatonin intake was 10.485 ± 13.306 μg/day. PSQI scores were positively correlated with NEQ scores (*r* = 0.537, *p* < 0.001) and negatively correlated with MEQ scores (*r* = −0.159, *p* = 0.004). In the multiple linear regression model, NEQ score was positively associated with PSQI score (*B* = 0.198, standardized *β* = 0.491, 95% CI: 0.156–0.239, *p* < 0.001), whereas MEQ score was negatively associated with PSQI score (*B* = −0.067, standardized *β* = −0.189, 95% CI: −0.102 to −0.032, *p* < 0.001). Female sex and chronic disease presence were also statistically significant, whereas total and evening dietary melatonin intake were not associated with PSQI score after adjustment for the measured covariates. In the logistic regression model for night eating syndrome risk, PSQI score (OR = 1.328, 95% CI: 1.185–1.488, *p* < 0.001) and MEQ score (OR = 0.941, 95% CI: 0.897–0.988, *p* < 0.001) were statistically significant. The exploratory indirect-association analysis identified a statistically significant indirect association among chronotype, night eating behaviors, and sleep quality.

**Conclusion:**

Eveningness tendency and higher night eating scores were associated with poorer sleep quality, whereas estimated dietary melatonin intake was not associated with sleep quality after adjustment for the measured covariates. These findings should be interpreted as cross-sectional and hypothesis-generating. Longitudinal and intervention studies are needed to clarify temporal relationships and to determine whether chronotype-informed or chrononutrition-based approaches influence sleep-related outcomes.

## 1 Introduction

Melatonin is a neurohormone synthesized in living organisms, primarily in the pineal gland of mammals, and plays a fundamental role in the regulation of circadian rhythm (1). Its synthesis is controlled by the suprachiasmatic nucleus located in the hypothalamus and regulated through a neuroendocrine mechanism sensitive to the light–dark cycle (2). Endogenous melatonin refers to melatonin synthesized within the body, primarily by the pineal gland, under the regulation of the light–dark cycle. This physiological process should be distinguished from dietary melatonin exposure derived from food consumption and from pharmacological melatonin administered as an exogenous supplement. Although melatonin was initially considered to be exclusively of animal origin, it has now been identified in a wide range of living organisms, including plants, thereby opening a new field of interest in nutritional science (3). In addition to its antioxidant, anti-inflammatory, and immunomodulatory properties, melatonin is known to play a critical role in the regulation of the sleep–wake cycle (4). Consequently, melatonin supplements are widely used in the management of sleep disorders. In recent years, it has been suggested that melatonin obtained through natural food sources may also exert physiological effects (5). However, the limited bioavailability of dietary melatonin and the substantial interindividual variability in circulating melatonin levels continue to raise uncertainty regarding whether these effects occur directly or through indirect mechanisms (6). Sleep is an essential biological process that significantly influences physical, cognitive, and emotional functioning (7). Stress, exposure to artificial light, and irregular dietary habits associated with modern lifestyles contribute to impaired sleep quality and disruption of circadian rhythm regulation (8). Therefore, the consumption of melatonin-rich foods has emerged as a non-pharmacological and potentially sustainable strategy for supporting sleep health (9, 10). Chronotype, which reflects interindividual differences in circadian rhythm, is an important determinant of biological time preference and is generally classified as morning type, evening type, or intermediate type (10). Individuals with an evening chronotype have been reported to be more prone to sleep disturbances and irregular eating behaviors (11). Night eating syndrome (NES), an important behavioral condition associated with chronotype, is characterized by excessive energy intake during the evening hours and nocturnal eating episodes following awakenings from sleep (12). NES has been shown to be closely associated with circadian rhythm disturbances and poor sleep quality (13, 14). Although the consumption of melatonin-rich foods has been proposed to exert beneficial effects on sleep quality and circadian regulation, most existing studies have focused on specific age groups, and the findings remain inconsistent (2, 9). In particular, studies examining dietary melatonin intake together with chronotype and night eating behavior in adults are limited. This highlights the need to evaluate dietary melatonin not only from a biochemical perspective but also in relation to behavioral and circadian dimensions.

Therefore, the present study aimed to evaluate the relationship between dietary melatonin intake and sleep quality, the presence of night eating syndrome, and chronotype characteristics in adults. In this context, the associations between dietary melatonin intake and sleep quality, as well as between evening consumption of melatonin-rich foods and symptoms of night eating syndrome, were investigated. Additionally, differences in dietary melatonin intake according to chronotype characteristics were examined.

## 2 Materials and methods

### 2.1 Study population and data collection

This cross-sectional study was conducted in Istanbul, Türkiye, between November 2024 and June 2025. The study used a convenience sampling method to recruit eligible adults living in Istanbul who voluntarily agreed to participate. The sample size was calculated using the G*Power 3.1.9.7 software. The calculation was based on multiple linear regression analysis. According to the analysis, assuming a small effect size (*f*^2^ = 0.05), a significance level of 5% (*α* = 0.05), and a statistical power of 80% (1 − *β* = 0.80), the required sample size was determined to be 309 participants. The study was ultimately completed with a total of 325 participants (15, 16). Ethical approval was obtained on October 30, 2024 (approval number: 61351342/020-533). The study was conducted in accordance with the principles of the Declaration of Helsinki.

The study population consisted of adults aged 18–65 years living in Istanbul who voluntarily agreed to participate and completed all questionnaires and data collection procedures. Participants were recruited through voluntary participation from different districts of Istanbul. Individuals receiving nutrition counseling at the measurement center were not included in the study. Exclusion criteria included being younger than 18 years or older than 65 years of age, illiteracy, pregnancy or lactation, diagnosis of cognitive impairment, use of melatonin supplements, antidepressants, or sleep medications, and failure to provide written informed consent.

Research data were collected through face-to-face interviews using questionnaires administered by the researcher and anthropometric measurements performed according to standardized procedures. Sociodemographic information, including demographic characteristics, anthropometric measurements, general health status, and dietary habits, was obtained from each participant. Anthropometric measurements, including body weight, height, waist circumference, and hip circumference, were performed under standardized conditions at a private nutrition and diet counseling center. The center served solely as the standardized site for anthropometric measurements. All measurements were performed following standard measurement techniques (17). Based on these measurements, body mass index (BMI), waist-to-hip ratio, and waist-to-height ratio were calculated.

Sleep quality was evaluated using the Pittsburgh Sleep Quality Index (PSQI). Night eating behavior was assessed using the Night Eating Questionnaire (NEQ). Chronotype characteristics were determined using the Morningness–Eveningness Questionnaire (MEQ). In addition, all participants completed a modified Food Frequency Questionnaire (FFQ) designed to evaluate the consumption of melatonin-containing foods, as previously described.

### 2.2 Data collection instruments

#### 2.2.1 Anthropometric measurements

##### 2.2.1.1 Body weight

Body weight (kg) was measured on a flat and stable surface using a calibrated digital scale (Tanita BC 730) with a sensitivity of 100 g and a maximum capacity of 150 kg. Participants were measured barefoot and wearing light clothing. All measurements were recorded in the questionnaire form (18).

##### 2.2.1.2 Height

Height (cm) was measured using a non-elastic measuring tape while participants stood upright against a wall with their feet together and their heels, hips, and head touching the wall. The head was positioned according to the Frankfurt plane. Measurements were recorded in the questionnaire form (18).

##### 2.2.1.3 Body mass index (BMI)

Body mass index (BMI) was calculated by dividing body weight (kg) by the square of height in meters (kg/m^2^). BMI classifications were evaluated according to the World Health Organization criteria (18, 19).

##### 2.2.1.4 Waist and hip circumference

Waist circumference was measured at the midpoint between the lower margin of the last rib and the upper border of the iliac crest. Hip circumference was measured while participants were standing upright, with the researcher positioned in front of the participant. The measurement was taken at the level of the greater trochanters of the femur by placing the measuring tape parallel to the floor. All measurements were recorded in the questionnaire form (19).

#### 2.2.2 Pittsburgh Sleep Quality Index (PSQI)

Sleep quality was assessed using the Pittsburgh Sleep Quality Index (PSQI), originally developed by Buysse et al. (20). The Turkish validity and reliability study of the scale was conducted by Ağargün et al. (21). The questionnaire consists of 24 items, of which 19 are self-reported by the participant and five are answered by a spouse or roommate. However, the total score is calculated solely based on the self-report items.

The PSQI evaluates sleep characteristics over the previous month and comprises seven components: subjective sleep quality, sleep latency, sleep duration, habitual sleep efficiency, sleep disturbances, use of sleep medication, and daytime dysfunction. The total score derived from these components provides an overall assessment of an individual’s sleep quality. Each component is scored between 0 and 3 points, resulting in a total score ranging from 0 to 21 (22).

In the present study, PSQI scores were categorized according to the cut-off value recommended in the literature. A total PSQI score of ≤5 was considered indicative of good sleep quality, whereas a score of >5 indicated poor sleep quality. The Cronbach’s alpha reliability coefficient of the PSQI was found to be 0.740 in this study.

#### 2.2.3 Chronotype assessment

Chronotype was assessed using the Morningness–Eveningness Questionnaire (MEQ), originally developed by Horne and Östberg (23) to evaluate individuals’ preferences regarding sleep–wake rhythms. The Turkish validity and reliability study of the scale was conducted by Pündük et al. (24). The questionnaire consists of self-report items assessing participants’ preferred sleep and wake times, daily performance levels, and the periods of the day during which they feel most energetic. Participants were asked to respond to the questions based on their usual daily routines.

The MEQ is a 19-item self-report scale designed to evaluate daily behavioral patterns according to morningness–eveningness preferences. Most items are rated using 4- or 5-point Likert-type response options, and the scoring range differs across items. Specifically, items 3, 4, 5, 6, 7, 8, 9, 13, 14, 15, and 16 are scored between 1 and 4 points; items 1, 2, 10, 17, and 18 between 1 and 5 points; items 11 and 19 between 0 and 6 points; and item 12 between 0 and 5 points. According to the cut-off values recommended in the literature, individuals were classified as evening type (16–41 points), intermediate type (42–58 points), or morning type (59–86 points) (25).

In the present study, MEQ scores were evaluated both as continuous variables and as categorical classifications in the analyses. The Cronbach’s alpha reliability coefficient of the Morningness–Eveningness Questionnaire was found to be 0.713 in this study.

#### 2.2.4 Night Eating Questionnaire

Night eating behavior was assessed using the Night Eating Questionnaire (NEQ), originally developed by Allison et al. (26). The Turkish validity and reliability study of the scale was conducted by Atasoy et al. (27). This 14-item self-report questionnaire evaluates morning appetite, evening and nocturnal eating behaviors, nocturnal awakenings with eating, food cravings, difficulty initiating sleep, and mood-related characteristics associated with night eating behavior.

The first nine items of the questionnaire are answered by all participants. Participants reporting nocturnal awakenings complete items 10–12, whereas those exhibiting night eating behavior respond to items 13 and 14. With the exception of item 7, most items are scored on a 5-point Likert scale ranging from 0 to 4. Item 7 assesses diurnal mood variation, and individuals reporting no mood change receive a score of 0. Items 1, 4, and 14 are reverse scored. Item 13 evaluates awareness of night eating behavior and is not included in the total score calculation.

The total NEQ score ranges from 0 to 52, with scores of 25 or higher indicating a risk for Night Eating Syndrome (28).

#### 2.2.5 Assessment of dietary melatonin intake

To evaluate participants’ dietary melatonin intake, a modified Food Frequency Questionnaire (FFQ), adapted from the dietary assessment forms used by Borisenkov et al. (6, 9), was administered. The modified FFQ was used to estimate habitual dietary melatonin intake by assessing the consumption frequency and portion sizes of melatonin-containing foods. Participants were asked to report the frequency and amount of consumption of these foods according to their usual dietary habits over the previous month. Dietary melatonin intake was calculated by combining food-specific melatonin concentration values reported in the literature with conversion coefficients corresponding to reported consumption frequencies, and daily intake was expressed as μg/day. Melatonin concentrations were assigned primarily according to the methodology described by Borisenkov et al. and supplemented with data from Grao-Cruces et al. (6, 9, 29). Foods were further classified into five melatonin-content categories: high, above average, average, below average, and low, in accordance with the approach proposed by Borisenkov et al. Portion sizes used in the dietary melatonin calculations were also adapted from Borisenkov et al. Detailed information on the foods included in the modified FFQ, assigned melatonin concentrations, melatonin-content categories, portion sizes, and literature references is provided in Supplementary Table S1. The modified FFQ was adapted from previously published dietary assessment forms used in dietary melatonin research. Therefore, no additional pilot testing or reproducibility assessment was performed in the present study. Since validated correction factors for cooking-related changes in melatonin content are currently unavailable, no adjustments were applied for cooking methods. Furthermore, the estimated dietary melatonin intake represents an approximation of habitual dietary exposure rather than a direct measure of systemic melatonin availability.

During the calculation process, the melatonin contents of foods reported in the literature were taken as reference values. Daily dietary melatonin intake was calculated for each food item using the reported consumption frequency, portion size, number of portions consumed, and assigned melatonin concentration. The reported consumption frequency category was converted into a frequency coefficient, which was multiplied by the consumed portion size and the assigned melatonin concentration of the food item. The values obtained for all food items were summed to estimate total daily dietary melatonin intake. Participants were asked to identify the foods they consumed from the listed products and to answer three main questions for each item.

1. How frequently did you consume these foods during the past month?
  Response options (with corresponding conversion coefficients for consumption frequency) were as follows: never (0), 1–2 times/month (0.05), 3–4 times/month (0.12), 2–3 times/week (0.36), 4–6 times/week (0.71), 1–2 times/day (1.5), 3–4 times/day (3.5), and more than 4 times/day (5).
2. How many portions of these foods did you consume in one meal?
  This question was accompanied by visual aids illustrating portion sizes and the gram weight of each product. Response options included 0.5, 1, 2, 3, 4, or 5 portions.
3. What percentage of the above foods was consumed during dinner?
  Response options were 0, 25, 50, 75%, or 100%.

Participants were also asked to report the average daily amount of each selected food item directly in grams. Accordingly, the portion amounts consumed per meal were additionally calculated.

#### 2.2.6 Statistical analysis

Statistical analyses were performed using SPSS Statistics version 25.0 (IBM Corp., Armonk, NY, USA). Descriptive data were presented as mean ± standard deviation, median (minimum–maximum), number, and percentage. The distribution of continuous variables was evaluated using skewness and kurtosis values. Differences between female and male participants were analyzed using the independent-samples *t*-test. Relationships between categorical variables were evaluated using the chi-square (*χ*^2^) test, and correlations between continuous variables were examined using Pearson correlation analysis.

Multiple linear regression analysis was performed to evaluate factors associated with sleep quality, as assessed by the PSQI total score. The primary model included age, sex, body mass index (BMI), waist circumference, smoking status, alcohol consumption, chronic disease presence, chronotype score, night eating score, total dietary melatonin intake, and evening dietary melatonin intake. Covariates were selected according to their conceptual relevance to sleep quality, chronotype, night eating behaviors, and dietary exposure, as well as their availability in the study dataset.

Although BMI and waist circumference represent related anthropometric indicators, both were retained in the model because they reflect general and central adiposity, respectively. Multicollinearity was evaluated using variance inflation factor values, which were within acceptable limits. Nevertheless, the potential conceptual overlap between these anthropometric indicators was considered when interpreting the findings. The assumptions of multiple linear regression were evaluated by examining linearity, normality and homoscedasticity of residuals, independence of errors, and multicollinearity. Independence of errors was assessed using the Durbin–Watson statistic. Unstandardized coefficients, standardized *β* coefficients, 95% confidence intervals, and *p* values were reported.

Binary logistic regression analysis was conducted to evaluate factors associated with night eating syndrome risk, defined as an NEQ score ≥25. Odds ratios (ORs), 95% confidence intervals (CIs), and *p* values were reported. Logistic regression model fit was assessed using the omnibus test, Hosmer–Lemeshow goodness-of-fit test, Cox and Snell *R*^2^, and Nagelkerke *R*^2^. Several potentially relevant factors, including physical activity, detailed caffeine intake, psychological distress, depressive and anxiety symptoms, socioeconomic conditions, occupational and shift-work schedules, evening artificial-light exposure, habitual sleep duration, and overall dietary quality, were not comprehensively assessed and therefore could not be included in the multivariable models.

In addition, an exploratory cross-sectional indirect-association analysis was performed using Hayes’ PROCESS macro Model 4 to examine the statistical indirect association among chronotype, night eating behaviors, and sleep quality. Bootstrap confidence intervals were estimated using 5,000 resamples. Given the cross-sectional design, all regression and indirect-association analyses were interpreted as association-based analyses. The PROCESS model was considered an exploratory statistical decomposition of cross-sectional covariance rather than evidence of temporal ordering or behavioral, biological, or causal mediation. Because multiple statistical comparisons were performed and no formal multiplicity correction was applied, findings close to the conventional significance threshold were interpreted cautiously. Statistical significance was accepted at *p* < 0.05.

## 3 Results

As presented in Table 1, most participants were women (74.5%), university graduates (65.5%), single (61.8%), and employed (58.5%). Educational level showed no statistically significant difference between sexes (*p* = 0.051). However, employment status differed significantly by sex, with men having a higher employment rate than women (86.7% vs. 48.8%, *p* < 0.001). Marital status also differed significantly between sexes, and the proportion of married participants was higher among men than among women (50.6% vs. 33.9%, *p* = 0.008). Women reported a higher prevalence of chronic disease than men; however, this difference did not reach statistical significance (23.6% vs. 13.3%, *p* = 0.062). Regular vitamin/mineral supplement use was significantly more common among women than men (20.7% vs. 2.4%, *p* < 0.001). In contrast, smoking prevalence was significantly higher among men than women (22.9% vs. 12.4%, *p* = 0.019). Alcohol consumption and regular medication use did not differ significantly between sexes (both *p* > 0.05). Chronotype distribution did not differ significantly by sex (*p* = 0.774). Overall, 92 participants (28.3%) were classified as evening type, 177 (54.5%) as intermediate type, and 56 (17.2%) as morning type. Poor sleep quality was observed in 54.8% of the participants, while NES risk was identified in 13.2%. Both poor sleep quality and NES risk were more frequent among women than men; however, the differences were not statistically significant (*p* > 0.05).

**Table 1:** Sociodemographic and lifestyle characteristics of the participants according to sex (*n* = 325).

Variables	Women (*n* = 242) *n* (%)	Men (*n* = 83) *n* (%)	Total (*n* = 325) *n* (%)	*p*-value*
Educational level				0.051
Primary–middle school	16 (6.6)	5 (6.0)	21 (6.5)	
High school	75 (31.0)	16 (19.3)	91 (28.0)	
University	151 (62.4)	62 (74.7)	213 (65.5)	
Employment status				<0.001
Employed	118 (48.8)	72 (86.7)	190 (58.5)	
Unemployed	118 (48.8)	9 (10.8)	127 (39.1)	
Retired	6 (2.5)	2 (2.4)	8 (2.5)	
Marital status				0.008
Married	82 (33.9)	42 (50.6)	124 (38.2)	
Single	160 (66.1)	41 (49.4)	201 (61.8)	
Presence of chronic disease				0.062
Yes	57 (23.6)	11 (13.3)	68 (20.9)	
No	185 (76.4)	72 (86.7)	257 (79.1)	
Regular medication use				0.338
Yes	33 (13.6)	8 (9.6)	41 (12.6)	
No	209 (86.4)	75 (90.4)	284 (87.4)	
Regular vitamin/mineral supplement use				<0.001
Yes	50 (20.7)	2 (2.4)	52 (16.0)	
No	192 (79.3)	81 (97.6)	273 (84.0)	
Smoking status				0.019
Yes	30 (12.4)	19 (22.9)	49 (15.1)	
No	212 (87.6)	64 (77.1)	276 (84.9)	
Alcohol consumption				0.692
Yes	18 (7.4)	5 (6.0)	23 (7.1)	
No	224 (92.6)	78 (94.0)	302 (92.9)	
Chronotype, *n* (%)				
Evening type (16–41 points)	66 (27.3)	26 (31.3)	92 (28.3)	0.774
Intermediate type (42–58 points)	134 (55.4)	43 (51.8)	177 (54.5)	
Morning type (59–86 points)	42 (17.4)	14 (16.9)	56 (17.2)	
Poor sleep quality, *n* (%)				
PSQI ≤5	102 (42.1)	45 (54.2)	147 (45.2)	0.057
PSQI >5	140 (57.9)	38 (45.8)	178 (54.8)	
NES risk, *n* (%)				0.263
NEQ < 25	207 (85.5)	75 (90.4)	282 (86.8)	
NEQ ≥ 25	35 (14.5)	8 (9.6)	43 (13.2)	

*p-values were calculated using the *χ*^2^ test. PSQI, Pittsburgh Sleep Quality Index; NEQ, Night Eating Questionnaire; NES, night eating syndrome.

As shown in Table 2, anthropometric measurements, including height, waist circumference, body weight, hip circumference, BMI, waist-to-hip ratio, and waist-to-height ratio, were significantly higher in men than in women (all *p* < 0.001). Although men were slightly older than women, the difference in age was not statistically significant (*p* = 0.060). Women had significantly higher PSQI scores than men, indicating poorer sleep quality among female participants (6.01 ± 2.86 vs. 4.70 ± 2.81, *p* < 0.001). In contrast, MEQ scores and NEQ scores did not differ significantly between sexes (both *p* > 0.05). Regarding dietary melatonin intake, total dietary melatonin intake was significantly higher in men than in women (12.503 ± 14.278 vs. 9.793 ± 12.914 μg/day, *p* = 0.004). However, evening melatonin intake did not differ significantly between sexes (*p* = 0.053; Table 2).

**Table 2:** Anthropometric measurements, sleep characteristics, and melatonin intake according to sex (*n* = 325).

Variables	Sex	Mean ± SD	Median	Min–Max	*t*-value	*p*-value
Height (cm)	Women	162.91 ± 5.61	163.00	150.00–183.00	−19.474	<0.001*
	Men	177.10 ± 6.04	177.00	164.00–196.00		
	Total	166.54 ± 8.43	165.00	150.00–196.00		
Waist circumference (cm)	Women	78.65 ± 8.99	79.00	52.00–110.00	−13.482	<0.001*
	Men	95.23 ± 11.43	93.00	50.00–145.00		
	Total	82.89 ± 12.06	82.00	50.00–145.00		
Body weight (kg)	Women	62.74 ± 10.94	60.00	43.00–101.00	−15.966	<0.001*
	Men	86.07 ± 12.97	85.00	60.00–145.00		
	Total	68.70 ± 15.34	65.00	43.00–145.00		
Hip circumference (cm)	Women	96.97 ± 8.22	95.00	80.00–125.00	−6.514	<0.001*
	Men	103.78 ± 8.25	104.00	85.00–130.00		
	Total	98.71 ± 8.74	98.00	80.00–130.00		
BMI (kg/m^2^)	Women	23.63 ± 3.91	22.70	16.85–41.50	−7.771	<0.001*
	Men	27.40 ± 3.49	27.08	18.83–42.37		
	Total	24.60 ± 4.14	24.14	16.85–42.37		
Waist-to-hip ratio	Women	0.81 ± 0.08	0.82	0.58–1.05	−9.682	<0.001*
	Men	0.92 ± 0.09	0.92	0.57–1.20		
	Total	0.84 ± 0.10	0.85	0.57–1.20		
Waist-to-height ratio	Women	0.48 ± 0.06	0.48	0.31–0.67	−7.362	<0.001*
	Men	0.54 ± 0.06	0.53	0.30–0.78		
	Total	0.50 ± 0.06	0.50	0.30–0.78		
Age (years)	Women	31.22 ± 10.50	28.00	18.00–63.00	−1.885	0.060
	Men	33.73 ± 10.48	31.00	18.00–61.00		
	Total	32.60 ± 10.55	30.00	18.00–63.00		
Sleep Quality Score	Women	6.01 ± 2.86	6.00	0.00–16.00	3.614	<0.001*
	Men	4.70 ± 2.81	5.00	0.00–15.00		
	Total	5.67 ± 2.90	5.00	0.00–16.00		
Chronotype Scale Score	Women	52.24 ± 7.71	53.00	33.00–73.00	0.363	0.717
	Men	51.87 ± 9.35	52.00	28.00–69.00		
	Total	52.15 ± 8.15	52.00	28.00–73.00		
Night Eating Questionnaire Score	Women	16.08 ± 7.27	15.00	1.00–41.00	1.469	0.143
	Men	14.73 ± 6.95	13.00	3.00–33.00		
	Total	15.74 ± 7.21	14.00	1.00–41.00		
Total melatonin intake	Women	9.793 ± 12.914	5.699	0.294–105.408	−2.873	0.004*
	Men	12.503 ± 14.278	8.272	0.761–101.828		
	Total	10.485 ± 13.306	6.316	0.094–105.408		
Evening melatonin intake	Women	0.99993 ± 2.38068	0.44495	0.00–28.53116	−1.939	0.053
	Men	0.94310 ± 1.05467	0.65142	0.00–6.36287		
	Total	0.98542 ± 2.12082	0.49770	0.00–28.53116		

*Independent samples *t*-test; statistically significant at *p* < 0.05.

Table 3 presents the multiple linear regression analysis for PSQI total score. The model was statistically significant (*F* = 17.91, *p* < 0.001), with *R*^2^ = 0.362 and adjusted *R*^2^ = 0.347. NEQ score was associated with PSQI total score (*B* = 0.198, standardized *β* = 0.491, 95% CI: 0.156–0.239, *p* < 0.001). MEQ score was also associated with PSQI total score (*B* = −0.067, standardized *β* = −0.189, 95% CI: −0.102 to −0.032, *p* < 0.001). Female sex (*B* = 0.984, standardized *β* = 0.162, 95% CI: 0.340–1.628, *p* = 0.003) and chronic disease presence (*B* = 0.931, standardized *β* = 0.127, 95% CI: 0.199–1.663, *p* = 0.013) were statistically significant. Total dietary melatonin intake and evening dietary melatonin intake were not statistically significant.

**Table 3:** Multiple linear regression analysis for sleep quality (PSQI total score).

Variables	Unstandardized *B*	SE	Standardized *β*	t	*p*-value	95% CI	VIF	Model summary	Value
Constant	7.214	1.126	—	6.406	<0.001	4.999–9.429	—	*R*	0.602
Age (years)	−0.012	0.014	−0.041	−0.857	0.392	−0.040 to 0.016	1.184	*R* ^2^	0.362
Sex (female)	0.984	0.327	0.162	3.009	0.003	0.340–1.628	1.231	Adjusted *R*^2^	0.347
BMI (kg/m^2^)	−0.031	0.028	−0.058	−1.104	0.271	−0.087 to 0.025	1.452	Standard Error of Estimate	2.331
Waist circumference (cm)	0.018	0.011	0.094	1.624	0.105	−0.004 to 0.040	2.316	F	17.91
Smoking status (yes)	0.742	0.418	0.088	1.775	0.077	−0.081 to 1.565	1.107	Model *p*-value	<0.001
Alcohol consumption (yes)	0.493	0.563	0.041	0.876	0.382	−0.614 to 1.600	1.063	Durbin–Watson	1.94
Chronic disease presence	0.931	0.372	0.127	2.503	0.013	0.199–1.663	1.219		
MEQ score	−0.067	0.018	−0.189	−3.722	<0.001	−0.102 to −0.032	1.284		
NEQ score	0.198	0.021	0.491	9.354	<0.001	0.156–0.239	1.173		
Total melatonin intake (μg/day)	0.009	0.007	0.061	1.238	0.217	−0.005 to 0.023	1.421		
Evening melatonin intake (μg/day)	−0.014	0.041	−0.016	−0.351	0.726	−0.094 to 0.066	1.367		

The dependent variable was the PSQI total score, with higher scores indicating poorer sleep quality. The model included age, sex, BMI, waist circumference, smoking status, alcohol consumption, chronic disease presence, MEQ score, NEQ score, total dietary melatonin intake, and evening dietary melatonin intake. Unstandardized coefficients (*B*), standardized coefficients (*β*), 95% confidence intervals, and variance inflation factor (VIF) values are reported. Higher MEQ scores indicate greater morningness, whereas higher NEQ scores indicate more pronounced night eating behaviors. Sex was coded as female versus male, and smoking and alcohol consumption were coded as yes versus no. BMI, body mass index; CI, confidence interval; MEQ, Morningness–Eveningness Questionnaire; NEQ, Night Eating Questionnaire; PSQI, Pittsburgh Sleep Quality Index; SE, standard error; VIF, variance inflation factor.

As shown in Table 4, in the model examining NES risk, PSQI score (OR = 1.328, 95% CI: 1.185–1.488, *p* < 0.001) and MEQ score (OR = 0.941, 95% CI: 0.897–0.988, *p* = 0.012) were statistically significant. In the model examining poor sleep quality, female sex (OR = 2.675, 95% CI: 1.394–5.132, *p* = 0.003), chronic disease presence (OR = 2.537, 95% CI: 1.220–5.279, *p* = 0.013), MEQ score (OR = 0.935, 95% CI: 0.903–0.969, *p* < 0.001), and NEQ score (OR = 1.219, 95% CI: 1.169–1.270, *p* < 0.001) were statistically significant.

**Table 4:** Binary logistic regression models for night eating syndrome risk and poor sleep quality.

Dependent variable	Predictor	*B*	SE	Wald *χ*^2^	OR	95% CI	*p*-value
Night eating syndrome risk (NEQ ≥ 25)	Age (years)	−0.021	0.017	1.521	0.979	0.946–1.014	0.217
	Sex (female)	0.288	0.451	0.407	1.334	0.551–3.229	0.523
	BMI (kg/m^2^)	0.058	0.039	2.197	1.060	0.982–1.144	0.138
	Waist circumference (cm)	0.019	0.012	2.404	1.019	0.996–1.044	0.121
	Smoking status (yes)	0.641	0.391	2.685	1.899	0.882–4.090	0.101
	Alcohol consumption (yes)	0.577	0.538	1.149	1.781	0.621–5.107	0.284
	Chronic disease presence	0.503	0.371	1.836	1.654	0.799–3.425	0.175
	PSQI score	0.284	0.058	23.927	1.328	1.185–1.488	<0.001
	MEQ score	−0.061	0.024	6.347	0.941	0.897–0.988	0.012
	Total melatonin intake (μg/day)	−0.003	0.011	0.069	0.997	0.975–1.019	0.792
	Evening melatonin intake (μg/day)	0.084	0.076	1.231	1.087	0.936–1.263	0.267
Poor sleep quality (PSQI >5)	Sex (female)	0.984	0.332	8.759	2.675	1.394–5.132	0.003
	Chronic disease presence	0.931	0.374	6.207	2.537	1.220–5.279	0.013
	MEQ score	−0.067	0.018	13.862	0.935	0.903–0.969	<0.001
	NEQ score	0.198	0.021	87.726	1.219	1.169–1.270	<0.001

Odds ratios represent associations adjusted for the measured covariates included in each model and should not be interpreted as fully independent or causal effects. Model summary: For night eating syndrome risk: −2 Log Likelihood = 186.441; Cox & Snell *R*^2^ = 0.211; Nagelkerke *R*^2^ = 0.284; Hosmer–Lemeshow *χ*^2^ = 5.971, *p* = 0.641; classification accuracy = 84.3%; overall model *χ*^2^ = 48.526, *p* < 0.001. For poor sleep quality: omnibus test *χ*^2^ = 41.882, *p* < 0.001; Hosmer–Lemeshow *χ*^2^ = 6.214, *p* = 0.514.

BMI, body mass index; CI, confidence interval; MEQ, Morningness–Eveningness Questionnaire; NEQ, Night Eating Questionnaire; NES, night eating syndrome; OR, odds ratio; PSQI, Pittsburgh Sleep Quality Index; SE, standard error.

**Figure 1:**
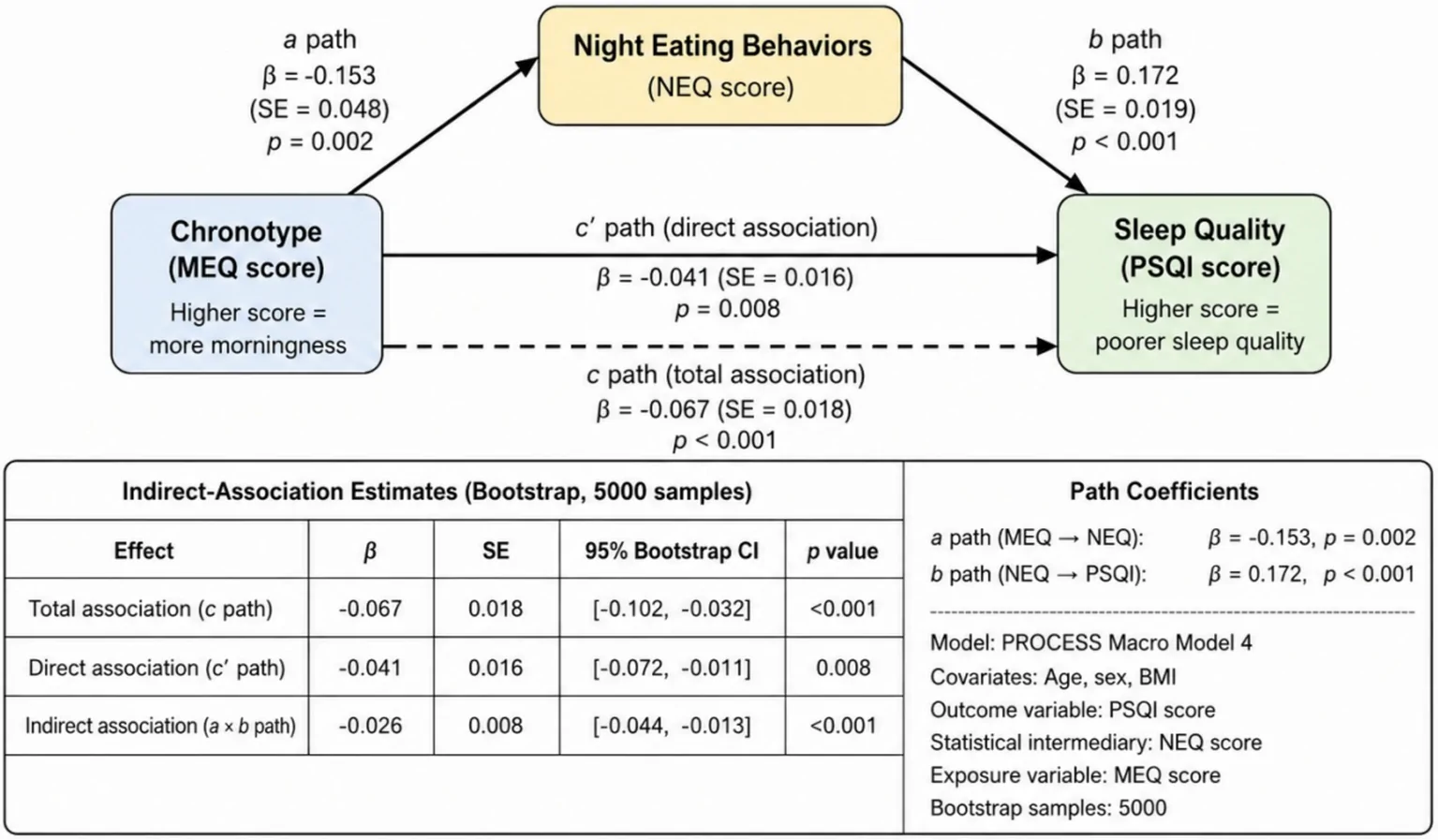
Exploratory cross-sectional indirect association among chronotype, night eating behaviors, and sleep quality. MEQ, Morningness–Eveningness Questionnaire; NEQ, Night Eating Questionnaire; PSQI, Pittsburgh Sleep Quality Index; SE, standard error; CI, confidence interval.

**Figure 2:**
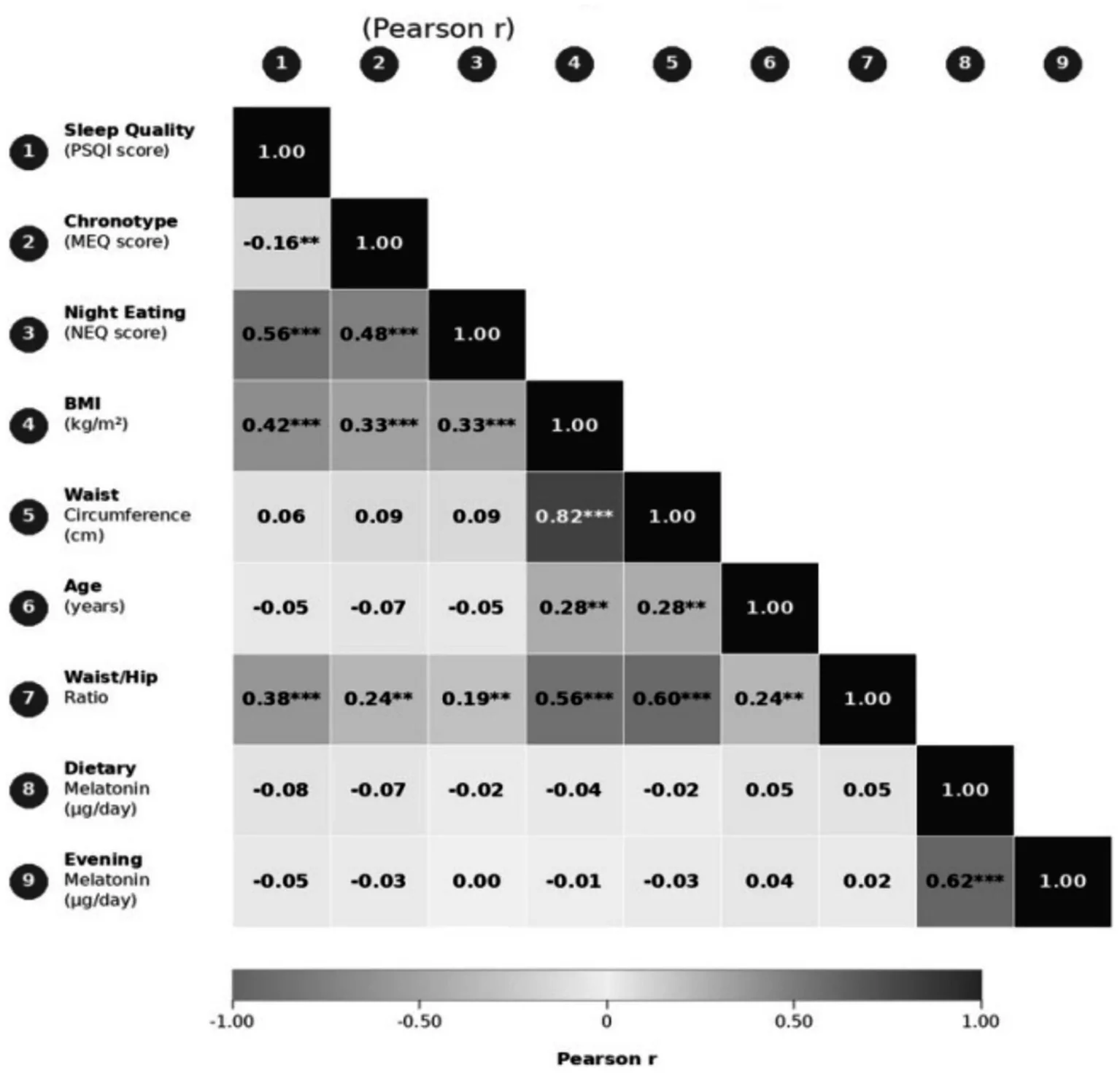
Correlation heatmap of key variables. Pearson correlation coefficients are presented. The PSQI–MEQ correlation was corrected to match the reported result (*r* = −0.159). Positive values indicate direct associations; negative values indicate inverse associations. Variables: (1) PSQI, (2) MEQ, (3) NEQ, (4) BMI, (5) waist circumference, (6) age, (7) waist/hip ratio, (8) dietary melatonin, (9) evening melatonin.

**Figure 3:**
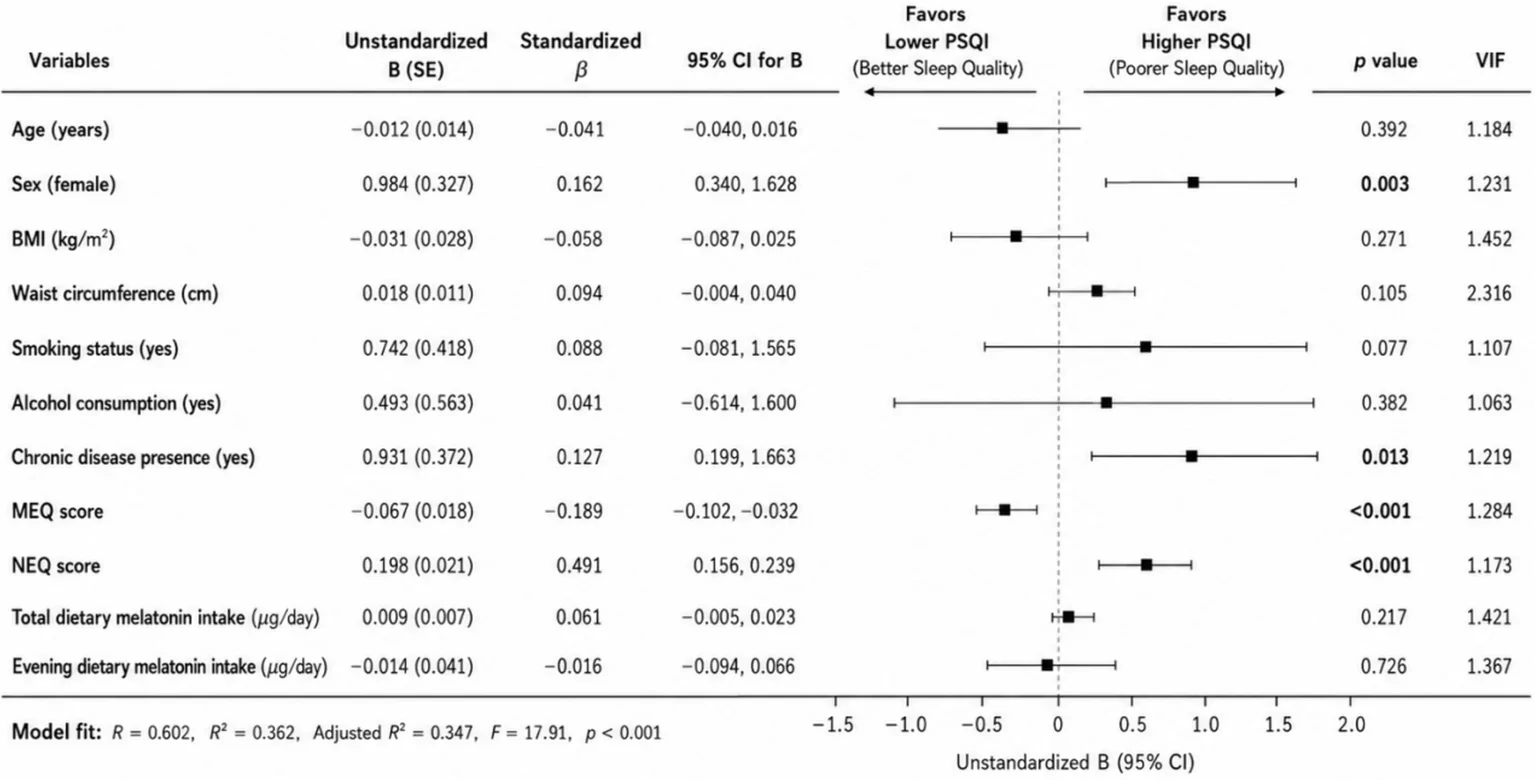
Forest plot of factors associated with sleep quality (multiple linear regression analysis for PSQI total score). Points represent unstandardized regression coefficients (*B*), and horizontal lines represent 95% confidence intervals. Higher PSQI scores indicate poorer sleep quality. PSQI, Pittsburgh Sleep Quality Index; MEQ, Morningness–Eveningness Questionnaire; NEQ, Night Eating Questionnaire; *B*, unstandardized regression coefficient; SE, standard error; *β*, standardized regression coefficient; CI, confidence interval, VIF, variance inflation factor. Positive *B* values indicate higher PSQI scores (poor sleep quality).

As shown in Figures 1–3, the visual analyses complemented the main statistical findings. Figure 1 presents the exploratory cross-sectional indirect-association model among chronotype, night eating behaviors, and sleep quality. Figure 2 summarizes the correlations among the principal study variables, while Figure 3 displays the standardized regression coefficients and corresponding 95% confidence intervals for factors associated with PSQI total score.

## 4 Discussion

This cross-sectional study examined the associations among dietary melatonin intake, chronotype, night eating behaviors, and sleep quality in adults living in Türkiye. More than half of the participants had poor sleep quality according to the PSQI cut-off. Eveningness tendency and higher night eating scores were associated with poorer sleep quality after adjustment for the measured covariates, whereas estimated dietary melatonin intake was not associated with sleep quality or NES risk. Overall, the findings indicate that behavioral and circadian characteristics were more closely related to sleep quality than estimated intake of a single dietary compound.

The absence of an association between dietary melatonin intake and sleep-related outcomes may have several explanations. Mean dietary melatonin intake in this sample was substantially lower than the levels proposed to exert physiological effects and far below pharmacological doses. Similar low habitual intakes have been reported in previous studies (30). In addition, food-derived melatonin may have limited or variable bioavailability, and intake estimates derived from food composition data may not reflect circulating melatonin exposure. Moreover, any sleep-related benefits of melatonin-rich foods may result from the combined effects of melatonin and other bioactive constituents rather than melatonin alone (31). Therefore, the null association observed in the present study should not be interpreted as definitive evidence of an absence of biological effect (32).

Habitual dietary melatonin intake should be clearly distinguished from pharmacological melatonin supplementation because these exposures differ substantially in dose, absorption, metabolism, and systemic availability. Pharmacological preparations generally provide melatonin in milligram doses, whereas habitual dietary exposure is usually in the microgram range and is distributed across multiple foods consumed throughout the day. Therefore, evidence derived from supplementation trials cannot be directly extrapolated to food-derived melatonin. In the present study, estimated dietary melatonin intake was not associated with sleep quality or NES risk after adjustment for the measured covariates, whereas chronotype and night eating behaviors remained associated with sleep-related outcomes. This finding suggests that habitual dietary melatonin exposure, at the levels observed in this sample, may not exert an effect comparable to that achieved through pharmacological supplementation. The results of supplementation studies should therefore be interpreted separately from observational estimates of dietary intake (33).

Several methodological and biological factors may also explain the absence of an association. Melatonin concentrations in foods are not constant and may vary according to cultivar., geographic origin, environmental growing conditions, stage of ripeness, storage duration, processing, and cooking method. As the present dietary estimate relied on published food-composition values, the assigned melatonin concentration may not have precisely represented the foods actually consumed by each participant. This variability is likely to have introduced non-differential exposure misclassification, which would tend to attenuate the observed associations. In addition, the use of a food frequency questionnaire may have introduced recall error in the reporting of consumption frequency and portion size. Although the questionnaire was adapted from previous dietary melatonin studies, it has not been validated against repeated dietary records or objective biomarkers in the present population. The bioavailability of food-derived melatonin represents another important source of uncertainty. The amount of melatonin present in a food does not necessarily correspond to the amount absorbed or reaching the systemic circulation. Digestion, intestinal absorption, first-pass metabolism, meal composition, and interindividual metabolic differences may all influence circulating melatonin concentrations after food consumption. Reviews of melatonin-containing foods have reported substantial heterogeneity in study designs and outcomes, while only a limited number of studies have confirmed changes in systemic exposure using biomarkers such as urinary 6-sulfatoxymelatonin (aMT6s) (34). Consequently, estimated dietary intake cannot be considered a direct indicator of endogenous melatonin secretion or circulating melatonin availability. Furthermore, melatonin-rich foods should not be interpreted as sources of an isolated bioactive compound. These foods also provide tryptophan, polyphenols, magnesium, B vitamins, and other antioxidant and anti-inflammatory constituents that may influence sleep through independent or synergistic mechanisms. For example, tryptophan contributes to serotonin and melatonin synthesis, whereas polyphenols and micronutrients may affect oxidative stress, inflammation, and neuroendocrine regulation (35). Therefore, any sleep-related benefit associated with melatonin-containing foods may reflect the combined effects of the overall food matrix rather than melatonin alone. This may partly explain why studies evaluating specific foods, such as milk or sour cherries, sometimes report beneficial sleep-related outcomes even though isolated estimates of dietary melatonin intake show inconsistent associations. Taken together, the null finding for dietary melatonin should not be interpreted as definitive evidence that food-derived melatonin has no biological relevance. Rather, it may reflect the relatively low exposure level in the present sample, uncertainty in food-composition values, incomplete knowledge of bioavailability, FFQ-related measurement error, and the difficulty of separating melatonin from other bioactive constituents of the foods consumed. Future studies combining detailed dietary assessment with objective biomarkers such as aMT6s or dim light melatonin onset may help determine whether food-derived melatonin contributes independently to sleep regulation.

Chronotype emerged as one of the main correlates of sleep quality in the present study. Higher MEQ scores, indicating greater morningness, were associated with lower PSQI scores, whereas eveningness tendency was associated with poorer sleep quality. This finding is consistent with previous studies reporting that evening chronotype is associated with poorer sleep quality, greater mood disturbance, and increased circadian misalignment (36–39). Juda et al. showed that late chronotypes, particularly among workers, experience shorter and poorer-quality sleep and greater social jetlag, while Van den Berg et al. linked eveningness with depressive symptoms and impaired sleep (36, 37). Similarly, evidence from daylight-saving time studies suggests that late chronotypes may adapt less effectively to externally imposed schedules, which may contribute to sleep disruption. Nevertheless, because chronotype and sleep quality were assessed concurrently in the present study, the observed association should be interpreted as cross-sectional rather than directional or causal.

The relationship between chronotype and sleep may also extend beyond sleep timing and involve chrononutrition-related behaviors. Morning-type individuals generally report earlier breakfast consumption, more regular meal timing, and fewer late eating episodes, whereas evening-type individuals are more likely to skip breakfast and consume meals later in the day (40–42). These behavioral differences may contribute to the association between eveningness tendency and poorer sleep quality through greater circadian misalignment and irregularity in daily routines. Although meal timing was not examined as a primary exposure in the present study, the coexistence of eveningness, higher night eating scores, and poorer sleep quality supports the relevance of considering chronotype and eating timing together. Longitudinal studies incorporating detailed meal-timing and sleep-timing assessments are needed to clarify the temporal relationships among these factors.

Night eating behaviors, reflected by higher NEQ scores, showed the strongest behavioral association with poorer sleep quality in the present study after adjustment for the measured covariates. This finding is consistent with the defining features of Night Eating Syndrome, including excessive evening intake, nocturnal eating episodes, morning anorexia, and disruption of the usual eating–sleep rhythm (13, 26). The exploratory indirect-association analysis also indicated that greater eveningness was associated with higher NEQ scores and that higher NEQ scores were associated with poorer sleep quality. However, because chronotype, night eating behaviors, and sleep quality were assessed concurrently, this pattern should be interpreted only as a statistical decomposition of cross-sectional covariance. It does not demonstrate that eveningness precedes night eating or that night eating subsequently causes poorer sleep.

Circadian misalignment may provide an additional framework for understanding the observed associations. Evening-type individuals may experience greater difficulty aligning their preferred sleep and activity timing with work and social schedules that are predominantly morning-oriented. This mismatch, commonly described as social jetlag, has been associated with irregular sleep timing and adverse metabolic outcomes (43, 44). In the present study, the association between greater eveningness and poorer sleep quality may therefore reflect not only chronotype itself but also incompatibility between biological timing and externally imposed schedules. Nevertheless, social jetlag and day-to-day sleep irregularity were not directly measured, and this interpretation remains hypothetical.

The relationship between sleep quality and eating behavior is also likely to be bidirectional. Sleep disturbance may promote later eating, greater energy intake, and less favorable dietary choices, whereas irregular or nocturnal eating may contribute to sleep disruption through behavioral, metabolic, or circadian mechanisms (45). Accordingly, the coexistence of eveningness, higher night eating scores, and poorer sleep quality should be interpreted within a broader chrononutrition framework rather than as evidence of a unidirectional pathway (46). In addition, unmeasured psychological, occupational, environmental, and lifestyle characteristics may have contributed to these associations; therefore, residual confounding cannot be excluded, and the magnitude of the observed relationships may have been partly overestimated.

### 4.1 Limitations and future directions

Several limitations should be acknowledged. The cross-sectional design precludes causal inference and temporal ordering among chronotype, night eating behaviors, dietary melatonin intake, and sleep quality; therefore, the mediation findings should be interpreted only as an exploratory statistical decomposition of cross-sectional associations. Self-reported data may have introduced recall and social desirability bias. Residual confounding cannot be excluded because physical activity, caffeine intake, psychological distress, depressive and anxiety symptoms, socioeconomic conditions, occupational and shift-work schedules, evening light exposure, habitual sleep duration, and overall dietary quality were not comprehensively assessed. In addition, because multiple statistical comparisons were performed without formal adjustment for multiplicity, the possibility of an inflated type I error rate cannot be excluded, particularly for findings close to the conventional significance threshold. Participants with chronic diseases or using vitamin/mineral supplements were not excluded. Dietary melatonin estimates may not fully reflect food preparation, variability in food melatonin content, or bioavailability. Finally, convenience sampling, the predominance of women, and recruitment of mainly urban adults may limit representativeness and generalizability. Future longitudinal and intervention studies incorporating objective circadian biomarkers are warranted.

## 5 Conclusions and clinical implications

In conclusion, this study identified significant cross-sectional associations among sleep quality, night eating behaviors, and chronotype. Eveningness tendency and higher night eating scores were more strongly associated with poorer sleep quality than estimated dietary melatonin intake, which was not associated with sleep quality after adjustment for the measured covariates. These findings support the joint consideration of chronotype and eating behaviors within a chrononutrition framework when examining sleep-related outcomes. However, the findings should be regarded as hypothesis-generating rather than as evidence supporting clinical recommendations. Longitudinal studies are needed to clarify temporal relationships, and randomized controlled trials are required to determine whether chronotype-informed or personalized chrononutrition interventions can improve sleep quality.

## Data Availability

The raw data supporting the conclusions of this article will be made available by the authors, without undue reservation.

## Supplementary material

Supplementary Table S1 Food-specific melatonin concentrations (FMT), categorized food melatonin (CFMT) values, and literature sources used for dietary melatonin estimation. Adapted from Borisenkov et al. (6) and Grao-Cruces et al. (29).
