# Supplementary material for: Dietary melatonin, chronotype, and night eating syndrome in relation to sleep quality among adults
Source: Front Nutr. 2026 Aug 13;13:1890075. doi: 10.3389/fnut.2026.1890075 (PMC13520769; doi:10.3389/fnut.2026.1890075)
Supplement: Supplementary file 1 [file Table_1.docx]

**Supplementary Table S1.** Food-specific melatonin concentrations (FMT), categorized food melatonin (CFMT) values, and literature sources used for dietary melatonin estimation

| Food product categories according to CFMT | CFMT value (ng/g fresh weight) | Food product | FMT concentration in food products, ng/g fresh weight | Reference |
| --- | --- | --- | --- | --- |
| 1.High | 100 | White mustard | 189.00 | Borisenkov et al., 2023 |
|  |  | Black mustard | 129.00 | Borisenkov et al., 2023 |
|  |  | Curcuma | 120.00 | Borisenkov et al., 2023 |
|  |  | Mulberry | 90.00 | Borisenkov et al., 2023 |
| 2.Above average | 10 | Soybean | 29.20 | Borisenkov et al., 2023 |
|  |  | Rice | 28.33 | Borisenkov et al., 2023 |
|  |  | Apple | 15.30 | Borisenkov et al., 2023 |
| 3.Average | 1 | Strawberry | 4.85 | Borisenkov et al., 2023 |
|  |  | Tomato | 4.45 | Borisenkov et al., 2023 |
|  |  | Salmon | 3.70 | Borisenkov et al., 2023 |
|  |  | Pomegranate | 3.02 | Borisenkov et al., 2023 |
|  |  | Cherry | 2.50 | Borisenkov et al., 2023 |
|  |  | Pork | 2.50 | Borisenkov et al., 2023 |
|  |  | Beef | 2.10 | Borisenkov et al., 2023 |
|  |  | Almond | 1.83 | Borisenkov et al., 2023 |
|  |  | Oat | 1.80 | Borisenkov et al., 2023 |
|  |  | Walnut | 1.77 | Borisenkov et al., 2023 |
|  |  | Chicken | 1.70 | Borisenkov et al., 2023 |
|  |  | Corn | 1.64 | Borisenkov et al., 2023 |
|  |  | Lamb | 1.60 | Borisenkov et al., 2023 |
|  |  | Egg | 1.54 | Borisenkov et al., 2023 |
|  |  | Apple juice | 1.43 | Borisenkov et al., 2023 |
|  |  | Ginger | 1.42 | Borisenkov et al., 2023 |
|  |  | Orange | 1.32 | Borisenkov et al., 2023 |
|  |  | Pine Nuts | 1.00 | Grao-Cruces et al., 2023 |
|  |  | Pistachios | 1.00 | Grao-Cruces et al., 2023 |
|  |  | Grape | 0.92 | Borisenkov et al., 2023 |
|  |  | Mango | 0.70 | Borisenkov et al., 2023 |
|  |  | White radish | 0.66 | Borisenkov et al., 2023 |
|  |  | Peanut | 0.60 | Grao-Cruces et al., 2023 |
|  |  | Pearl barley | 0.58 | Borisenkov et al., 2023 |
|  |  | Sunflower | 0.55 | Borisenkov et al., 2023 |
|  |  | Grape juice | 0.50 | Borisenkov et al., 2023 |
|  |  | Pumpkin seed | 0.50 | Grao-Cruces et al., 2023 |
| 4. Below average | 0.1 | Mango juice | 0.38 | Borisenkov et al., 2023 |
|  |  | Hazelnut | 0.35 | Borisenkov et al., 2023 |
|  |  | Pineapple | 0.30 | Borisenkov et al., 2023 |
|  |  | Banana | 0.28 | Borisenkov et al., 2023 |
|  |  | Carrot | 0.28 | Borisenkov et al., 2023 |
|  |  | Papaya | 0.24 | Borisenkov et al., 2023 |
|  |  | Cabbage | 0.21 | Borisenkov et al., 2023 |
|  |  | Pineapple juice | 0.20 | Borisenkov et al., 2023 |
|  |  | Cashew | 0.20 | Grao-Cruces et al., 2023 |
|  |  | Orange juice | 0.17 | Borisenkov et al., 2023 |
|  |  | Chinese cabbage | 0.11 | Borisenkov et al., 2023 |
|  |  | Olive oil | 0.10 | Borisenkov et al., 2023 |
|  |  | Pepper | 0.10 | Grao-Cruces et al., 2023 |
|  |  | beer | 0.01 | Grao-Cruces et al., 2023 |
|  |  | Onion | 0.09 | Borisenkov et al., 2023 |
|  |  | Cucumber | 0.09 | Borisenkov et al., 2023 |
|  |  | Cauliflower | 0.08 | Grao-Cruces et al., 2023 |
|  |  | Welsh onion | 0.09 | Borisenkov et al., 2023 |
|  |  | Sunflower oil | 0.06 | Borisenkov et al., 2023 |
|  |  | Peanut | 0.06 | Grao-Cruces et al., 2023 |
| 5.Low | 0.01 | Kiwifruit | 0.02 | Borisenkov et al., 2023 |
|  |  | Cow milk | 0.01 | Borisenkov et al., 2023 |
|  |  | Beetroot | 0.002 | Borisenkov et al., 2023 |

*FMT = Food Melatonin (Melatonin concentration in foods), CFMT = Categorized Food Melatonin (Categorized (rounded) food melatonin concentration)*
